# Composition Gradient Cellulose–Aerogel Nanocomposites Regulating Thermal Insulation

**DOI:** 10.1002/smsc.202300042

**Published:** 2023-08-23

**Authors:** Porus Sunil Jadhav, Arpita Sarkar, Shenqiang Ren

**Affiliations:** ^1^ Department of Materials Science and Engineering University of Maryland (UMD) College Park MD 20742 USA

**Keywords:** aerogel composites, functional gradient material, recycled cellulose, thermal insulation

## Abstract

Over the last few decades, functional gradient structures have evolved through natural biological systems, while gradient structures lead to tailored mechanical and physical performance due to the gradual structural change. Herein, composition gradient cellulose and aerogel nanocomposites that regulate their thermal insulation and mechanical performance are reported. The as‐prepared gradient composite shows a thermal conductivity of 32.2 mW m^−1^ K^−1^ and flexural modulus of 660 MPa while exhibiting superhydrophobicity and superior reusability. The unique orientation‐dependent thermal insulation and mechanical strength arise from the composition gradients formed by the silica aerogel distribution during the cellulose‐fiber‐percolated network formation, which opens a pathway toward green building thermal insulation materials.

## Graphical abstract

A gradient cellulose–aerogel nanocomposite is prepared by incorporating water glass‐based silica aerogel with recycled cellulose fiber networks, which shows low thermal conductivity, good mechanical strength, recyclability, and superhydrophobicity.

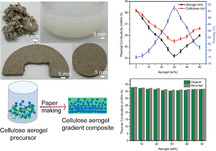

## 1 Introduction

Nature‐inspired functional materials with superior performance and improved sustainability have grown a large interest due to their wide range of applications, including energy absorption and generation, structural protection, and thermal engineering.^[^ 1, 2, 3 ^]^ One example of such nature‐inspired structures is the functional gradient materials.^[^ 4, 5, 6 ^]^ Functional gradient materials are materials with gradual variations in their structures and compositions throughout their volume and hence locally tailored properties.^[^ 7, 8, 9 ^]^ Functional gradient materials are usually found in nature such as plants (e.g., bamboo) and local tissue or bone in seashells.^[^ 10, 11, 12 ^]^ The compositions and architectures of functional gradient materials differ from isotropic bulk materials in several ways. Its microstructure can be designed to generate properties with specialized multifunctionality.^[^ 13, 14 ^]^ Moreover, the functional gradient structures are heterogeneous and they influence the mechanical and thermal properties of the composite to a great extent.^[^ ^15^ ^]^ Due to the mechanical and heat transfer transitions of the gradient structure, the microstructure produces a multiaxial stress state and a strain gradient which enhance the mechanical and thermal properties of the gradient structure.^[^ 16, 17, 18 ^]^

Nature‐inspired gradient structures can also be achieved using biogenic materials. Cellulose is an abundant natural polymer that can be obtained from agricultural wastes and recycled paper pulp.^[^ 19, 20, 21 ^]^ Cellulose extracted from nature has a huge carbon‐storing potential.^[^ ^22^ ^]^ However, the major drawbacks of using cellulose for thermal insulation are its limited thermal performance, mechanical strength, and hydrophilic characteristics.^[^ 23, 24, 25 ^]^ In this work, we report a nature‐inspired functional gradient aerogel–cellulose nanocomposite by incorporating silica aerogel with recycled cellulose fiber networks. The gradient structure composites display a consistent change in concentration of silica aerogel along the thickness direction, which dictates its thermal and mechanical properties. This gradient structure presents a morphology that significantly improves the mechanical strength of the composites while exhibiting thermal conductivity of 32.2 mW m^−1^ K^−1^, a flexural modulus of 660 MPa, porosity of 90%, hydrophobicity with a water contact angle of 110°, and recyclability with a recovery of 94%. This work represents a new methodology for the synthesis of thermal insulating, mechanically robust, and hydrophobic gradient composites.

## 2 Results and Discussion

**Figure 1:**
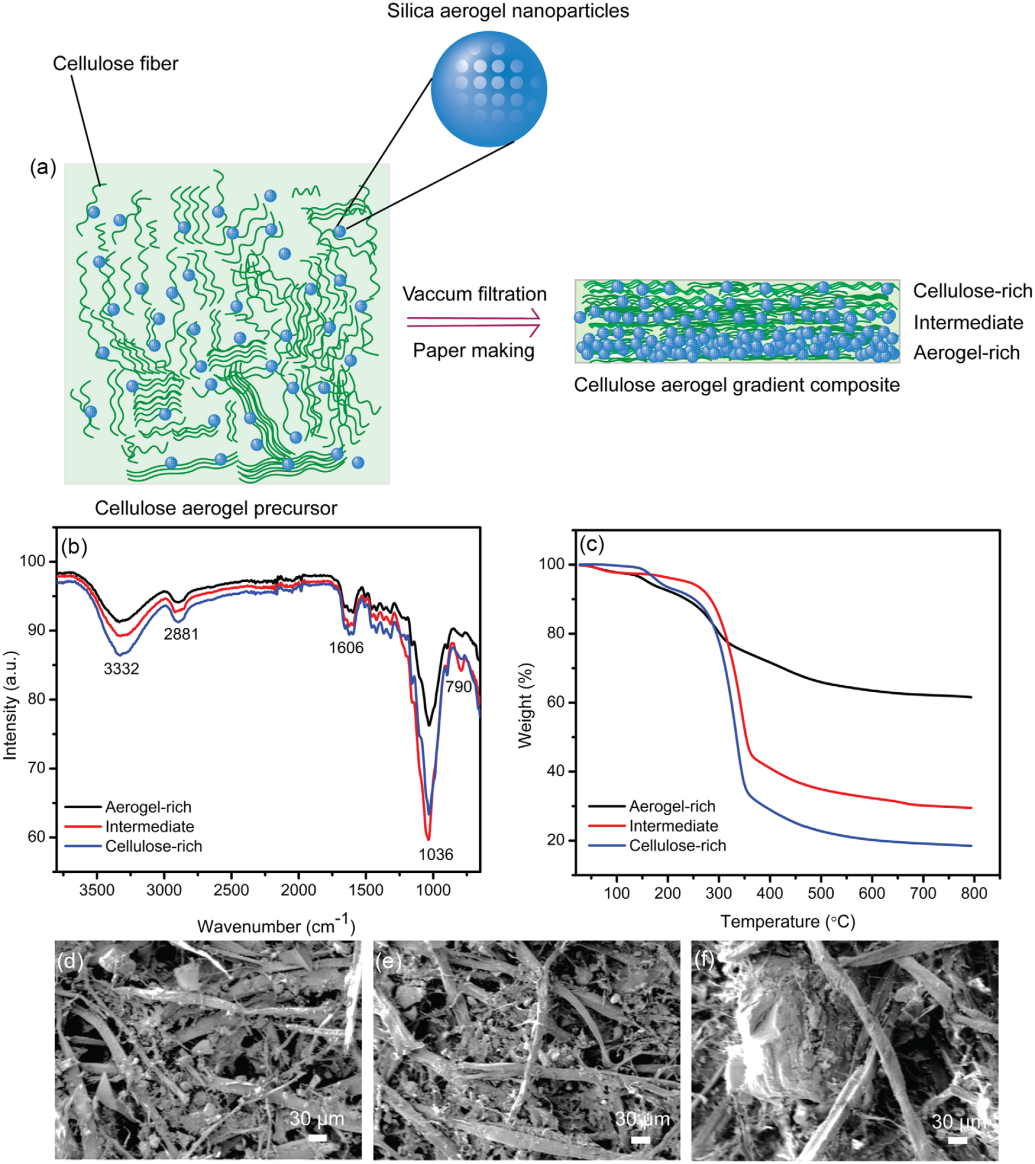
a) Schematic diagram for the synthesis of gradient cellulose–aerogel composite. b) FTIR spectra of different layers of the gradient composite. c) TGA curve of different layers of gradient composite. d–f) SEM–EDS images of the cellulose‐rich layer (d), the intermediate layer (e), and the aerogel‐rich layer of the gradient composite (f).

**Figure** **1**a shows the schematic manufacturing diagram of the functional gradient cellulose–silica‐aerogel composites. For the synthesis of the functional gradient composite, the cellulose fibers from the recycled paper pulp are mixed with silica aerogel which is then phase‐separated and interpenetrated into the cellulose fiber networks through the vacuum filtration for the formation of a functional gradient structure with respect to the silica aerogel concentration. Figure S1, Supporting Information, shows the images of the gradient and bilayer composites. To characterize gradient aerogel–cellulose composites, Fourier‐transform infrared (FTIR) spectroscopy is employed, as shown in Figure 1b. The different layers sliced from the gradient composites show typical absorptions of the cellulose backbone such as 3332 cm^−1^(υ_O–H_), 2881 cm^−1^ (υ_C–H_), 1429 cm^−1^ (υ_H–O–H_), 1036 cm^−1^ (υ_C–O–C_), and 896 cm^−1^ (υβ‐linkage).^[^ ^26^ ^]^ It is interesting to note that the intensity of the O–H stretching at 3332 cm^−1^ decreases with increasing aerogel concentrations in the aerogel‐rich layer, indicating the formation of a composition gradient structure. Moreover, a peak appearing around 790 cm^−1^ results from the Si–O vibrations, and an adjacent peak at approximately 970 cm^−1^ can be associated with rocking bending vibrations of Si–OH groups, indicating that silica is impregnated into the aerogel–cellulose composites. Figure S2, Supporting Information, shows the FTIR spectra of the bilayer composite which also displays similar characteristics from both silica aerogel and cellulose components. Furthermore, thermogravimetry analysis (TGA) is employed to study the functional gradient structure of the cellulose–aerogel composite. Figure 1c shows the comparisons of the TGA curve of different layers sliced from the gradient composite. The major weight loss observed in the temperature range between 350 and 400 °C due to the decomposition of cellulose. Moreover, the silica aerogel content from the aerogel‐rich layer, intermediate layer, and cellulose‐rich layer of the gradient composite is estimated at a weight percentage of 61%, 28%, and 18%. Figure 1d–f and S3, Supporting Information, illustrate the scanning electron microscopy (SEM) and energy‐dispersive X‐ray spectroscopy (EDS) mapping of three different layers, suggesting the distribution of silica aerogel into the cellulose fiber networks in the gradient composite. Figure S4, Supporting Information, represents the SEM images of the bilayer composite.

**Figure 2:**
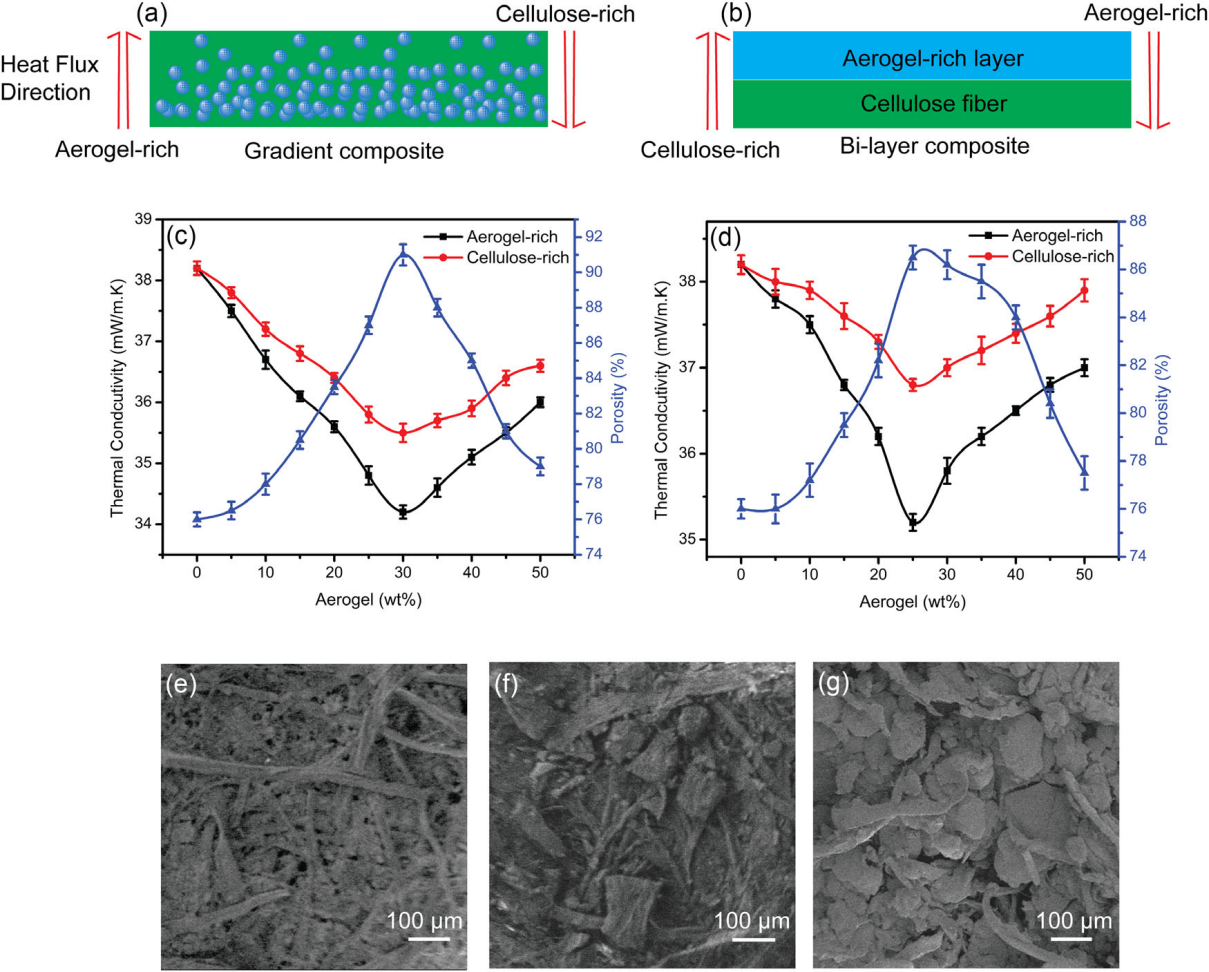
a,b) Schematic diagram of the heat flux direction for the gradient composite and bilayer composite. c,d) Thermal conductivity vs aerogel concentration variation for the gradient and bilayer composite, respectively. e) SEM images of the 5 wt% cellulose–aerogel gradient composite. f) SEM image of the 30 wt% cellulose–aerogel gradient composite. g) SEM image of the 50 wt% cellulose–aerogel gradient composite.

The thermal insulation behavior exhibits an orientation dependence in both functional gradient and bilayer composites. **Figure** **2**a,b represents the schematic diagram of heat flux direction for gradient and bilayer composites. Figure 2c shows a decrease in thermal conductivity of the gradient composites from 37.5 to 34.5 mW m^−1^ K^−1^ with an increase in the average aerogel concentration from 5 to 30 wt%, which is due to the high porosity and low density of the composites with increasing aerogel concentration resulting in lower thermal conductivity. However, when the average aerogel concentration is increased above 30 wt%, the thermal conductivity of the composite increases as a result of the aggregation of silica aerogel particles. A similar trend has been observed in the case of bilayer structure composites (Figure 2d). The SEM images (Figure 2e–g) reveal the aggregation of aerogel particles with increasing aerogel concentration in the gradient cellulose–aerogel composite. Another significant trend in the change of thermal conductivity is seen in the composites as the heat flows from different directions. Figure 2c,d describes the different orientations studied for gradient structure and bilayer structures. In the case of gradient structure, the difference in the thermal conductivity increases from (0.5 to 1.3 mW m^−1^ K^−1^) between the aerogel‐rich layer and cellulose‐rich layer of the composite (Figure 2c). Due to the gradient characteristics, silica aerogel accumulated in the bottom of the gradient composites results in a decreased thermal conductivity as the heat flow from the aerogel‐rich layer. A similar phenomenon is observed in the case of the bilayer composite. In the case of bilayer structure, the difference in thermal conductivity increases from 0.5 to 1.8 mW m^−1^ K^−1^ between the aerogel‐rich layer and cellulose‐rich layer as a result of the drastic thermal conductivity difference between silica aerogel and cellulose. Figure 2d shows the thermal conductivity of the bilayer structure which decreases from 37.7 to 35.2 mW m^−1^ K^−1^ with increasing the average aerogel concentration up to 25 wt%. It should be noted that the thermal conductivity and the density of the bilayer composite are higher than that of the gradient composites (Figure S5, Supporting Information).

**Figure 3:**
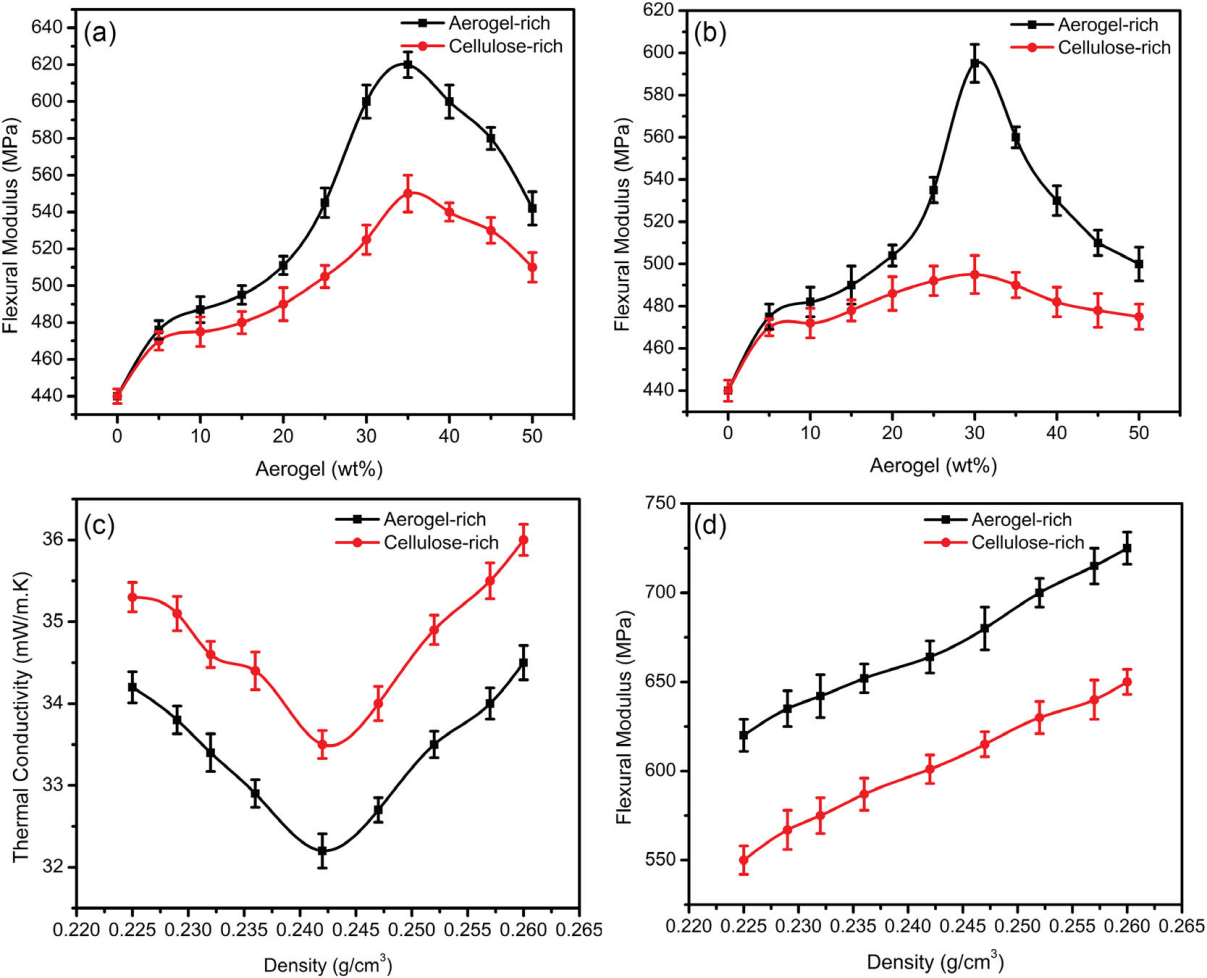
a) Flexural modulus versus aerogel (wt%) plot of gradient cellulose–aerogel composite. b) Flexural modulus vs aerogel (wt%) plot of bilayer cellulose–aerogel composite. c) Thermal conductivity vs density plot of gradient cellulose–aerogel composite. d) Flexural modulus vs density plot of gradient cellulose–aerogel composite.

The mechanical properties of the gradient composite could significantly affect the application of building insulation materials, while Figure S6, Supporting Information, shows the 3‐point bending test of the composites. **Figure** **3**a,b shows the flexural modulus for both gradient and bilayer composites. With the mechanical impact from the aerogel‐rich layer, the functional gradient composites show a higher flexural modulus than that of the cellulose‐rich layer. The difference in flexural modulus between the cellulose‐rich and aerogel‐rich layer of the gradient composite is from 10 to 80 MPa with increasing the average aerogel concentration from 35 to 50 wt%. For the bilayer structures, it shows a higher difference in flexural modulus from 10 to 100 MPa with increasing the average aerogel concentration from 5 to 30 wt%. Furthermore, we have also studied the thermal conductivity and flexural modulus change in the gradient structure with the density dependence. Figure 3c shows the change in density and thermal conductivity of the gradient composite while the density variation is achieved by using the pressure dependence during the processing. The thermal conductivity of the aerogel‐rich side of the gradient composite is reduced from (34.2 to 32.2 mW m^−1^ K^−1^) by changing the density from 0.225 to 0.242 g cm^−3^ and then it increases to 34.5 mW m^−1^ K^−1^ with changing the density to 0.26 g cm^−3^. At a lower density, the presence of air pockets in the cellulose fiber network structure leads to a high thermal conductivity. With increasing the density to an optimum, the silica aerogel and cellulose fiber networks show a decreased thermal conductivity. However, when the density is further increased, the composite is tightly packed and densified which can increase the heat transfer by the contacts between solids and increase thermal conductivity accordingly. Similar phenomena are observed in the case of mechanical properties with changing density (Figure 3d).

**Figure 4:**
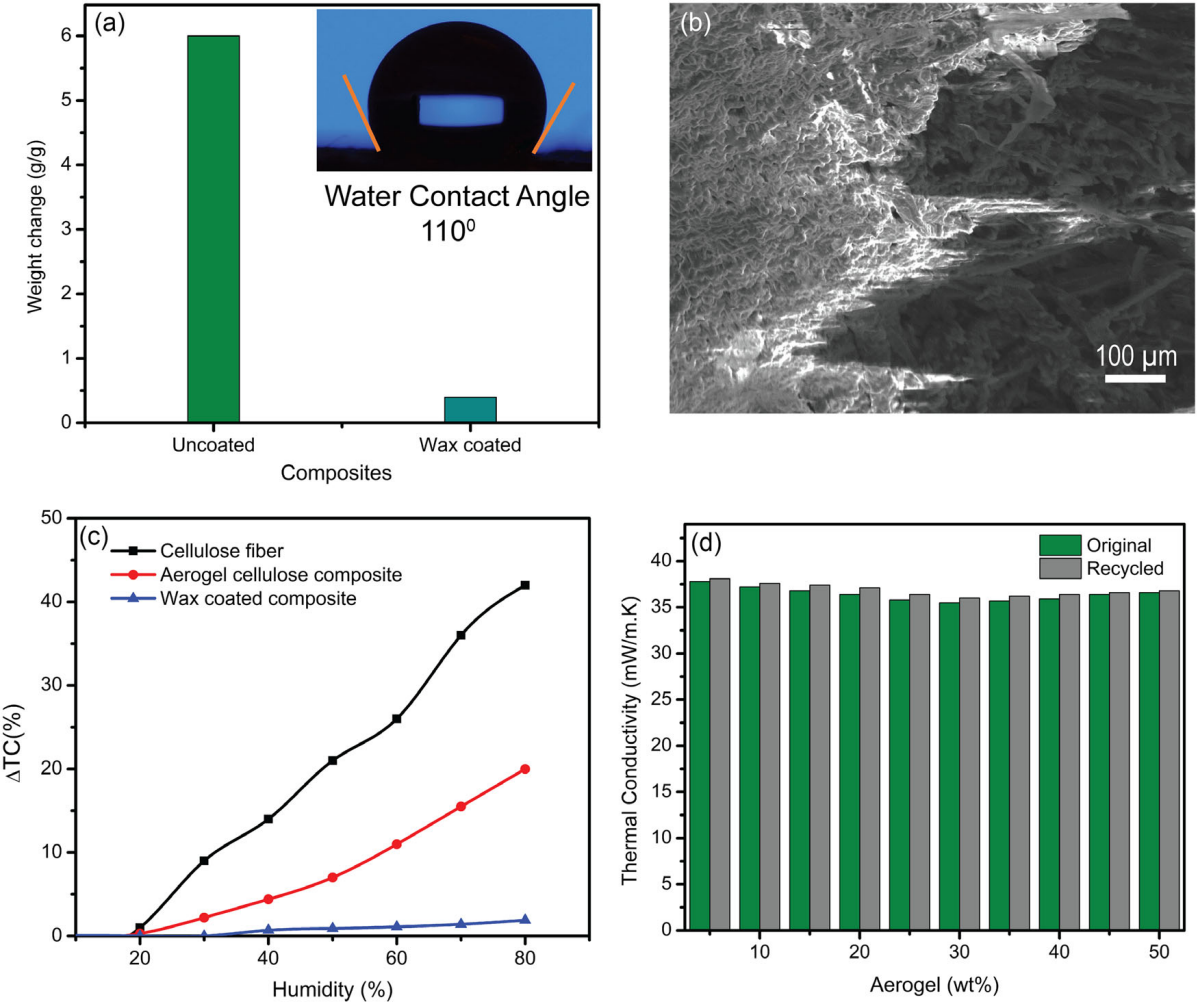
a) Change in water absorption capacity of gradient cellulose–aerogel composite and wax‐coated gradient cellulose–aerogel composite; inset water contact angle for the wax‐coated gradient composite. b) SEM image of wax‐coated gradient composite. c) Thermal conductivity vs humidity (%) plot of cellulose fiber, aerogel–cellulose gradient composite, and wax coated gradient composite. d) Reusability test of gradient cellulose–aerogel composites.

The water absorption capacity and wettability play an important role in using cellulose‐fiber‐based building applications due to their biogenic nature. **Figure** **4**a shows that in the case of the wax‐coated composite, the water absorption capacity is 8 times lower than that of the without‐coated composite, confirming the hydrophobic effect of the wax‐coated gradient composite on weatherability. Figure 4a (inset) shows the water contact angle of 110° in the wax‐coated composite. Figure 4b represents the surface morphology of the coated gradient composite which reveals the presence of wax coating layer over the surface of the composite. The humidity‐dependent thermal insulation study using a programmable humidity and temperature chamber is shown in Figure 4c. Figure 4c shows the comparison of the increment of thermal conductivity of cellulose fiber, with and without wax‐coated cellulose‐silica aerogel composite. The highest increase in thermal conductivity (≈28%) is observed in the case of cellulose fiber. The gradient composites without and with wax coating show a change of 10% and 3%, respectively, indicating hydrophobic coating role in its water absorption capacity. Moreover, the reusability and recyclability of the gradient composite, as shown in Figure 4d, represents the thermal conductivity of the original and recycled cellulose–aerogel gradient composites, on which its recovery percentage of 94% signifies the reusability and sustainability of the as‐prepared aerogel‐cellulose gradient composites.

## 3 Conclusion

We report gradient composites based on cellulose fiber and silica aerogel that regulate their thermal insulation and mechanical strength for building insulation applications. Due to the composition gradient in composites, this leads to tailored thermal insulation and mechanical properties while exhibiting orientation‐ and concentration‐dependent behaviors. The as‐prepared gradient composite shows a low thermal conductivity of 32.2 mW m^−1^ K^−1^, a flexural modulus of 660 MPa, porosity of 90%, and hydrophobicity with a water contact angle of 110°. The nature‐inspired gradient structure plays a significant role in carbon‐sequestration building thermal insulation applications.

## 4 Experimental Section

4.1

4.1.1

##### Materials

Sodium dodecyl sulfate, sodium silicate solution (water glass), and urea were purchased from Sigma Aldrich. Hydrogen chloride (HCl) was purchased from Fischer Scientific. The recycled cellulose fiber was obtained from the Clean Fiber. The hydrophobic coating was carried out using wax purchased from U.C. Coatings.

##### Synthesis of Sodium‐Silicate‐Based Aerogel Precursor

At first, 1 g sodium dodecyl sulfate and 9.0 g (0.15 mol) urea were mixed in 100 mL deionized (DI) water and stirred for 3 h at room temperature. Once the solution became homogenous, 11 mL of reagent‐grade sodium silicate solution was added to it and the solution mixture was kept on stirring for another 15 min. After that, 2 m HCl was slowly added to the mixture until the pH of the solution reached 9.0 and the solution was kept for overnight in a preheated oven at 60 °C at ambient pressure for gelation.

##### Purification Process

For the purification of the aerogel precursor first, the silica aerogel was taken into a 3 L beaker, and 2 L of DI water was added to it. After that, the aerogel mixture was homogenized using a mechanical agitator and then kept at 60 °C at ambient pressure for 24 h. Once a phase separation is visible the top water part is drained out without losing the gel part. This procedure was repeated for 4 times until the water part of the phase separation was not clear.

##### Synthesis of Gradient Cellulose–Aerogel Composite

First 20 g of blended clean fibers were taken and poured into a beaker. After that, 200 mL of silica aerogel was added to it. The amount of fibers and silica aerogel was varied to synthesize different wt% of gradient cellulose‐aerogel composite. Then, 3 L of DI water was added to the mixture for homogenization. A mechanical agitator was used for 30 min to homogenize the mixture. After homogenization, the mixture was poured into the paper‐making setup. Once the composite was made it was kept between metallic perforated sheets and these sheets were clamped using screws to maintain the thickness of the composite and to avoid buckling of the composite. Finally, the composite was kept in a preheated oven at 60 °C at ambient pressure for drying.

##### Synthesis of Bilayered Cellulose–Aerogel Composite

1) 17 g of blended clean fibers were taken into a beaker and 2 L DI water was added to it for homogenization. A mechanical agitator was used for 30 min to homogenize the fiber slurry and then it was poured into a paper‐making setup and the resulting wet composite membranes were transferred into a mold. 2) Simultaneously a homogenous mixture of 3 g of clean fiber and 200 mL of silica aerogel was prepared. Similar to the gradient structures, the amount of fibers and silica aerogel taken for the synthesis of the gradient composite was varied to get the various wt% of bilayered cellulose–aerogel composite. 1 L of DI water and mechanical agitator used for homogenization. This aerogel and fiber mixture was then poured above the previous panel into the mold and let the layers of the mixture settle for 1 h. Similar to the gradient structure, an assembly using screws and perforated sheets was made for bilayer structures. This assembly was kept in a preheated oven at 60 °C at ambient pressure for drying.

##### Physical Measurements

Finely ground composite samples were prepared to find the chemical bonding state and the interfacial bonding of composite synthesized at different concentrations and different layers and were examined using FTIR spectroscopy (FTIR‐Agilent carry 560). Microstructures of the composite structures were studied using a focused ion beam scanning electron microscope (FIB‐SEM, Carl Zeiss AURIGA Crossbeam).

A Thermtest heat flow meter 100 series (HFM‐100), which complies with ASTM C518 was used to calculate the thermal conductivity of the gradient, bilayer, and pure fiber samples. Equation (1) was used to calculate the bulk density of the gradient, bilayer, and pure fiber samples.

(1) $$\text{Density }\left(\rho \right)=\frac{\text{mass}(m)}{\text{volume}\left(v\right)}$$

Skeletal density (*ρ*_s_) was measured using a pycnometer system (Micromeritics Accu‐Pyc II 1340 Gas Pycnometer) which applies the gas displacement method to measure the volume and determine the density on a skeletal level. Porosity was calculated using Equation (2).

(2) $$\text{Porosity }\;=\;(1-\;\frac{\rho_{m}}{\rho_{s}})$$

Flexural modulus was used as a property to study the mechanical of the samples; uniaxial flexural modulus tests of specimens with bulk dimensions of 130 mm × 25 mm × 6 mm were carried out using a universal test system (Model SSTM‐20 KN from United Testing Systems) for testing samples up to 500 N.

## Conflict of Interest

The authors declare no conflict of interest.

## Supporting information

Supplementary Material

## Data Availability

The data that support the findings of this study are available in the supplementary material of this article.
