# Supplementary material for: Composition Gradient Cellulose–Aerogel Nanocomposites Regulating Thermal Insulation
Source: Small Sci. 2023 Aug 23;3(10):2300042. doi: 10.1002/smsc.202300042 (PMC11935866; doi:10.1002/smsc.202300042)
Supplement: Supplementary file 1 — Supplementary Material [file SMSC-3-2300042-s001.pdf]

Supporting Information

Composition Gradient Cellulose–Aerogel Nanocomposites Regulating Thermal Insulation

Porus Sunil Jadhav,[a,#] *Arpita Sarkar,*[a,#] *Shenqiang Ren* [a,*]

[a] Department of Mechanical and Aerospace Engineering, University at Buffalo (SUNY), Buffalo, NY 14260, USA

# Equal Contribution

* Email: shenren@buffalo.edu

Experimental methods:

The thermal conductivity of the aerogel-cellulose composites were measured using a Thermtest's Heat flow meter 100 series (HFM-100), which complies with the ASTM C518. A calibration file was used to set up the thermal conductivity measurements. The equipment measures the thermal conductivity of the composite by a steady-state methodology. Once the heat flow between the clamping plates and through the specimen has properly converged to a steady value over time, the system can determine the thermal conductivity of the material.

The thermal stability of the gradient composites is evaluated by means of thermogravimetric analysis and differential scanning calorimetry (TGA/DSC) tests carried out using the TA Instrument DSC SDT Q600. During TGA measurements, samples are heat-treated in a nitrogen atmosphere (with a purge rate of 100 mL/minute) from room temperature (~15 to 25 °C) to 800 °C with a heating rate of 10 °C/min.

The uniaxial 3-point bending tests of specimens with dimensions of 130mm x 25 mm and thickness in the region of 6 mm was carried out using a universal test system (Model SSTM-20KN from United Testing Systems). The composite pieces of the provided dimensions were loaded into a 3-point bending testing vice and an indenter was made to bend the sample in the center, with end conditions being max force of 500N or indenter position to reach 20mm in depth from the surface of the test sample. After that, the force and displacement data were recorded on the system. In the next step, the stress-strain curve was plotted, and flexural modulus was calculated. For every composite, four different samples were tested, and the average value was reported for accurate results.

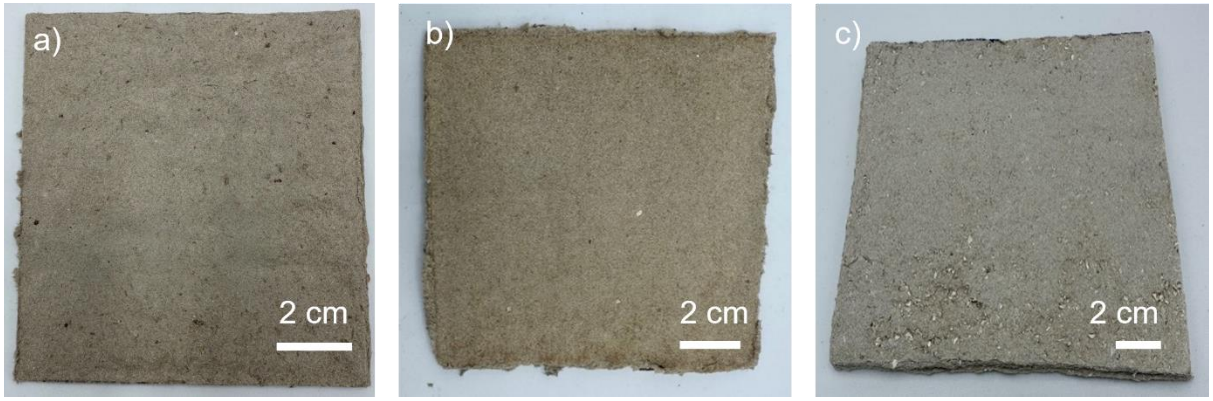

Figure S1. Images of a) pure fiber, b) gradient composite, and c) bi-layer composite.

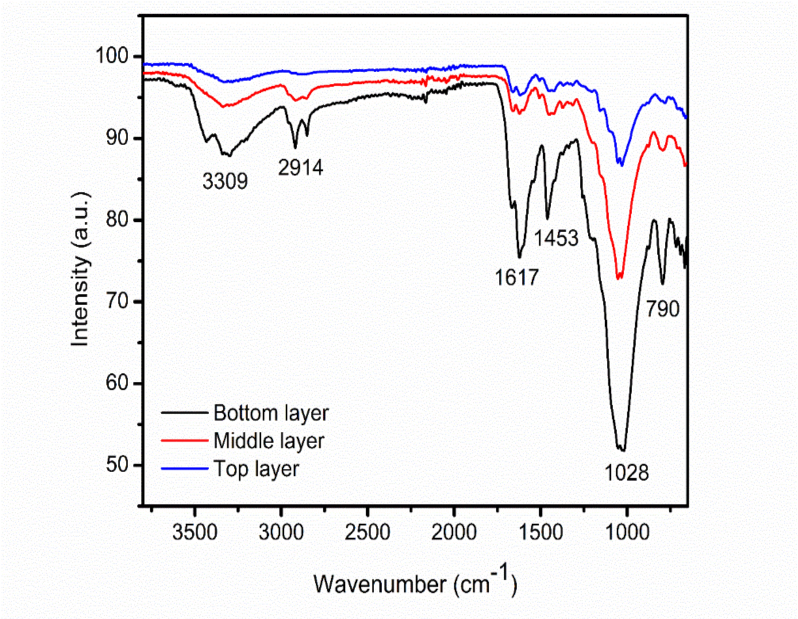

Figure S2. FTIR spectra of different layers of bi-layer composite.

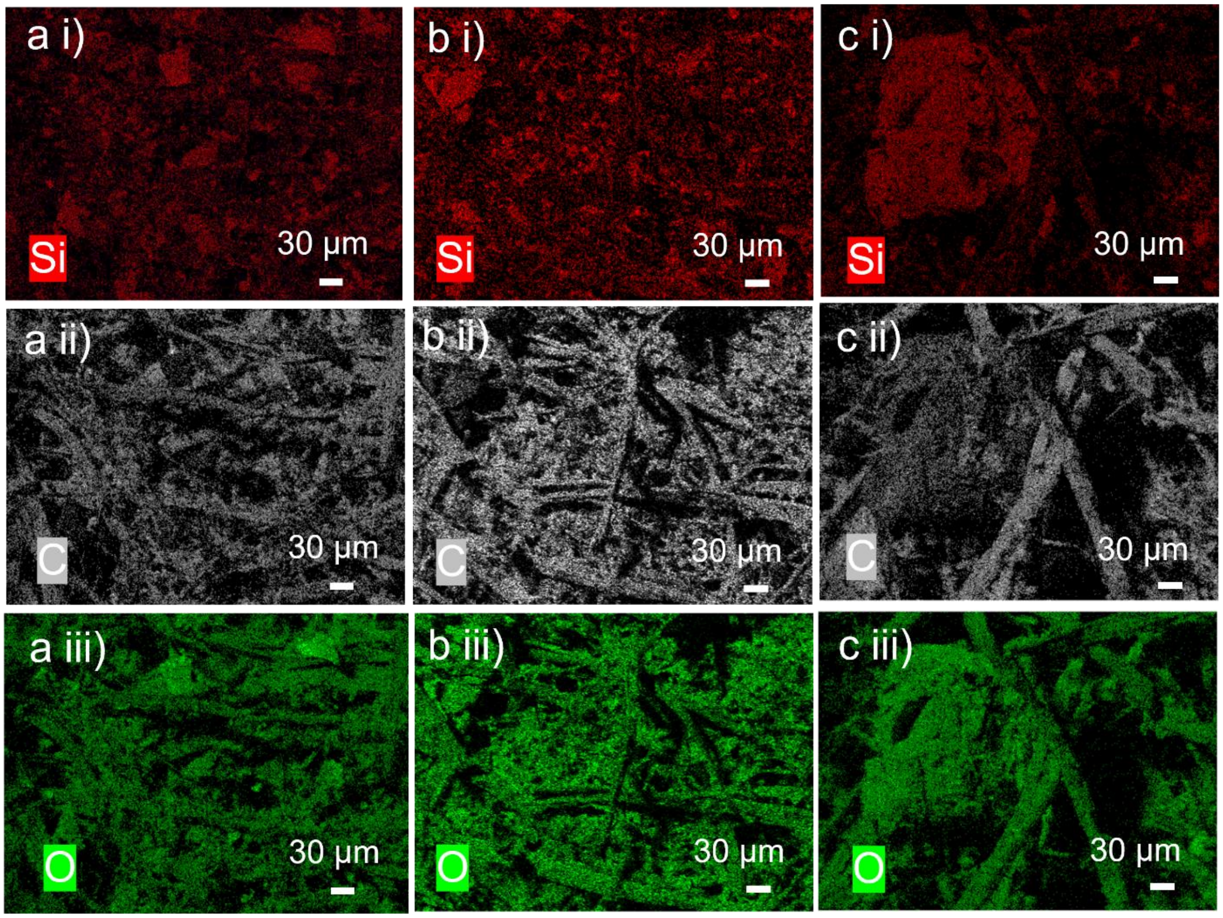

Figure S3. EDS mapping of a) cellulose-rich layer, b) intermediate layer, and c) aerogel-rich layer of gradient composite.

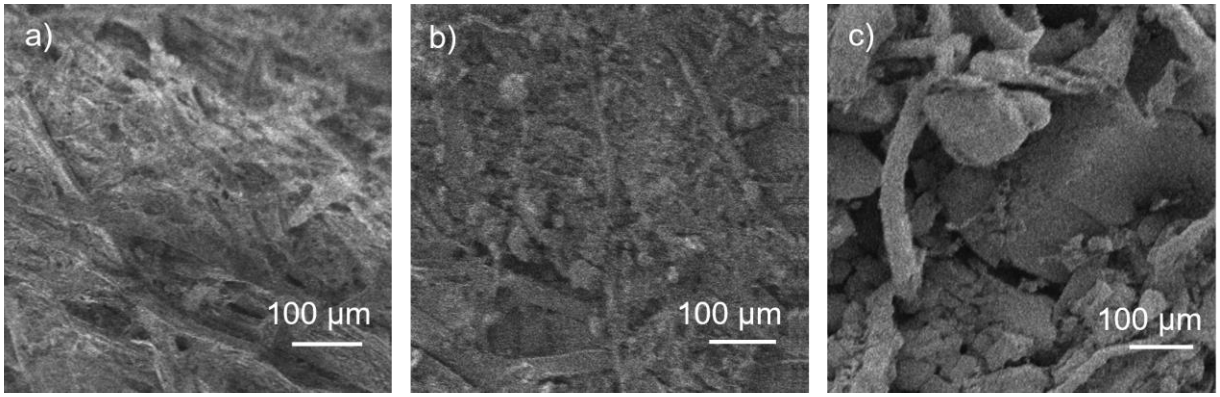

Figure S4. SEM images of a) cellulose-rich layer, b) intermediate layer, and c) aerogel rich layer of the bi-layer composite.

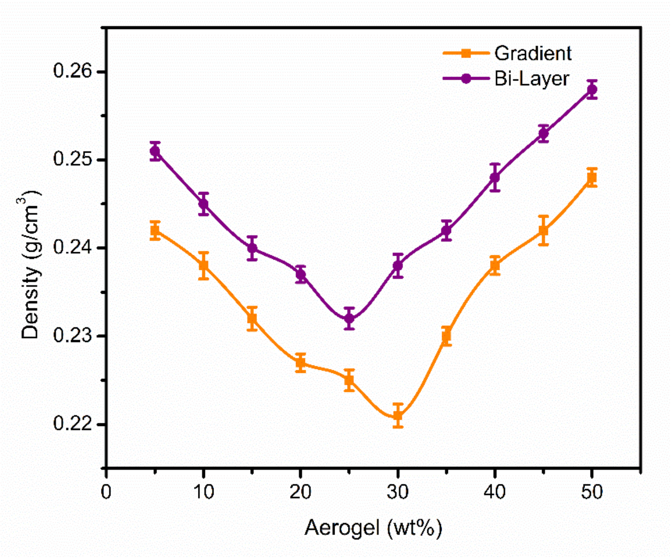

Figure S5. a) Density vs aerogel (wt%) plot of gradient and bilayer composite.

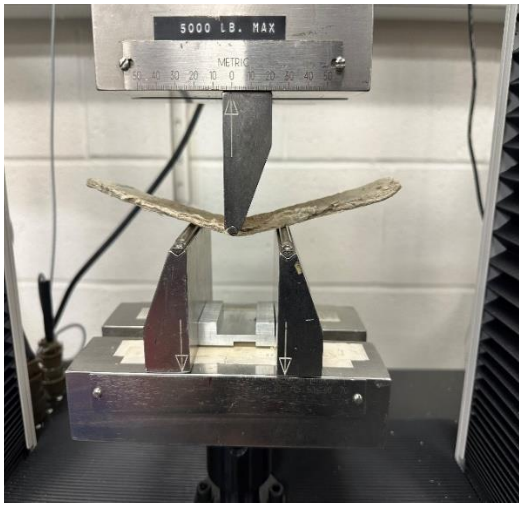

Figure S6. 3-point bending test of the composites.
